# Ecotoxicological impacts of caffeine on *Daphnia magna*: insights from acute to chronic exposures

**DOI:** 10.1007/s10646-025-02955-z

**Published:** 2025-08-29

**Authors:** Sara Rodrigues, Cláudia Gonçalves, Oksana Golovko, Sara C. Antunes

**Affiliations:** 1 Departamento de Biologia, Faculdade de Ciências da Universidade do Porto, Rua do Campo Alegre S/N, Porto, 4169-007 Portugal; 2 Centro Interdisciplinar de Investigação Marinha e Ambiental (CIIMAR/CIMAR), Terminal de Cruzeiros do Porto de Leixões, Avenida General Norton de Matos S/N, Matosinhos, 4550-208 Portugal; 3 Department of Aquatic Sciences and Assessment, Swedish University of Agricultural Sciences (SLU), Uppsala, SE-75007 Sweden

**Keywords:** Emerging contaminant, Psychoactive stimulant, Large waterflea, Behaviour, Biochemical endpoints, Reproductive parameters

## Abstract

Caffeine (CAF), a widely consumed central nervous system stimulant, has been detected in aquatic ecosystems, and several studies show that CAF affects non-target organisms. Although some ecotoxicological studies on CAF and aquatic organisms already exist, they mostly focus on marine organisms with concentrations relevant to those ecosystems. Thus, this study evaluated the ecotoxicological effects of CAF on *Daphnia magna* through four tests: acute exposure (48 h; 27–3000 mg/L; immobilization/mortality parameters), feeding inhibition assay evaluating immobilization/mortality parameters (24 h; 249–1000 mg/L), sub-chronic (10 days; 3.8–60 mg/L), and chronic (21 days; 4.6–35 mg/L) exposures, assessing life history parameters (age at first reproduction, fecundity, somatic growth rate, population growth rate, and reproductive output). In all assays, biochemical parameters like antioxidant defence biomarkers, lipid peroxidation, and cholinergic neurotransmission were evaluated. The results showed that CAF exposure caused feeding inhibition (EC_50_ = 323 mg/L), reduced somatic growth and fecundity (CAF ≥ 10 mg/L; *p* < 0.05), and induced changes in antioxidant defences (CAF ≥ 10 mg/L; *p* < 0.05) and acetylcholinesterase activity (CAF ≥ 60 mg/L; *p* < 0.05). These findings demonstrate that CAF induces toxic effects in *D. magna*, compromising key biological functions like growth, reproduction, and metabolic pathways, highlighting the need to monitor CAF residues found in aquatic environments. Moreover, these results offer critical data for environmental regulation, as the assessed parameters showed sensitivity in evaluating caffeine toxicity in freshwater organisms.

## Introduction

Caffeine (CAF) is an alkaloid (1,3,7-trimethylxanthine) that acts as a central nervous system stimulant, naturally occurring in various plants (e.g., *Camellia sinensis*, *Coffea arabica*, *Paullinia cupana*) (Barone et al., 1996). It is also an ingredient in several medications, cosmetics, aesthetic products, and foods like soft drinks, energy drinks, chocolates, snacks, and desserts (Rocha et al. 2022). For this reason, it has been considered one of the most widely consumed substances globally by humans (EFSA, 2015; Statista 2023) over the years. Based on global population estimates and average consumption, identified daily caffeine consumption in Europe ranging from 36.5 to 319.4 mg/day/adult between 1997 and 2015, with an average daily intake of 227 mg (Verster et al. 2017). Recent studies indicate that *per capita* caffeine consumption in Europe remains among the highest globally, with countries like the Netherlands, Finland, and Sweden leading the list (EFSA, 2015; Statista 2023). According to a 2023 report by the World Coffee Organization, Northern European countries, especially the Nordic nations, continue to lead Europe in per capita caffeine consumption (Temple et al. 2017; Statista 2023).

Caffeine can be synthetically produced and is considered a biologically active substance (Abdelkader et al. 2013; Vieira et al. 2022). Although this compound has been identified as a potential aquatic contaminant (Dafouz et al. 2018; Korekar et al. 2020; Vieira et al. 2022), it has not yet been included on the “Watch List” of the Water Framework Directive (WFD), as a substance to monitor in aquatic ecosystems (Gomez Cortes et al. 2022). Caffeine has high water solubility (20 g/L) and a long half-life ranging from 100 to 240 days (Vieira et al. 2022) up to 10 years (Edwards et al. 2018). Additionally, caffeine shows great stability under different conditions, such as variations in salinity, temperature, and light (Diogo et al. 2023a). Thus, the increasing consumption of CAF can have environmental repercussions (Li et al. 2019) due to the rising concentration in aquatic ecosystems. Indeed, CAF has already been detected in various aquatic matrices globally such as seawater 0.016–8.23 µg/L (Weigel et al. 2002; Vieira et al. 2022), wastewater treatment plants (WWTP) influents 2.88–3594 µg/L (Nodler et al. 2010; Adeleye et al. 2022), rivers/lakes and reservoirs 0.003–39.8 µg/L (Buerger et al., 2003; Cerveny et al. 2022), and WWTP and hospital effluents ranging from 0.02 to 86,000 µg/L (Nodler et al. 2010; Oliveira et al. 2015). Bruton et al. (2010) reported that the efficiency of WWTP in removing CAF is around 95%, however, this percentage can vary depending on the treatment methods used by the WWTP. Despite WWTPs showing relatively high efficacy rates in removing this compound, the growing global consumption has caused CAF concentrations in aquatic matrices to rise (Montagner et al. 2014), resulting in the increasing environmental detection of caffeine in various aquatic matrices (Korekar et al. 2020).

CAF is a psychoactive drug that can cause various pharmacological and biological effects in humans, but undesirable effects on aquatic species have already been reported (e.g., *Diopatra neapolitana*, *Hediste diversicolor*, *Danio rerio*) (Abdelkader et al. 2013; Faudone et al. 2021; Diogo et al. 2023b). This substance is classified as a non-selective antagonist of adenosine receptors (Cerveny et al. 2022) and stimulates the central nervous system, affecting neurotransmitter activity (Santos-Silva et al., 2018; Zhang et al. 2021). Some authors have already demonstrated the acute toxicity of CAF to several organisms, specifically by median lethal concentrations (LC_50_) and concentrations required to produce effects in 50% of the population (EC_50_), thus highlighting the growing concern in assessing CAF ecotoxicity (Bantle et al. 1994; Aguirre-Martínez et al. 2016; Pires et al. 2016; Korekar et al. 2020; Cerveny et al. 2022; Diogo et al. 2023a). Previous studies have reported LC_50_ and/or EC_50_ values ranging from LC_50_ (96 h) = 87 mg/L in fish (*Leuciscus idus*), algal growth inhibition (*Scenedesmus subspicatus*, EC_50_ (72 h) > 100 mg/L), as well as in growth inhibition of bacteria (*Pseudomonas putida*, EC_50_ (17 h) = 3490 mg/L) (Bantle et al. 1994; OECD 2012a; Farias et al. 2021). In *D. magna*, the reported EC_50_ (48 h; immobilization) was 182 mg/L (OECD 2012a), while in *Danio rerio* embryos, a 7‑day LC_50_ of 283.2 mg/L has been observed (Farias et al. 2021). Beyond acute effects, chronic exposures at environmentally relevant concentrations (ng/L to µg/L) have demonstrated reproductive impairments, behavioral alterations, and oxidative stress responses in aquatic invertebrates such *D. magna* and fish (Lu et al. 2013; Cruz et al. 2016; Korekar et al. 2020; Cerveny et al. 2022; Diogo et al. 2023a; Teixeira et al. 2025), reinforcing the need for long‑term ecotoxicological assessments of CAF.

Although several studies have assessed the ecotoxicological effects of CAF in aquatic organisms, most have focused on marine species or model fish, often under acute exposure scenarios, using concentrations higher than those typically found in freshwater environments. There is still limited knowledge on the sublethal and chronic effects of caffeine on freshwater invertebrates, particularly *D. magna*, which play a key role in aquatic food webs and is widely used as bioindicators. Furthermore, few studies have integrated individual- and sub‑individual‑level endpoints to elucidate the mechanisms underlying CAF toxicity. This gap hinders a comprehensive understanding of the ecological risks posed by caffeine in freshwater ecosystems. Therefore, the main objective of this study was to evaluate the biological responses of *D. magna* to CAF, through individual and sub-individual responses, after different exposure conditions (concentrations and exposure time). For this purpose, ecotoxicological assays were conducted to assess effects on mortality, swimming behavior, feeding rate, and life history traits (e.g., reproduction, growth) following acute, sub-chronic, and chronic exposures to CAF. Additionally, to assess sub-individual effects, biochemical markers involved in different metabolic pathways and physiological functions were quantified, specifically oxidative stress (catalase (CAT) and glutathione S-transferases (GSTs) activity), lipid peroxidation (levels of thiobarbituric acid reactive substances (TBARS)), and cholinergic neurotransmission (acetylcholinesterase (AChE) activity).

## Materials and methods

### Caffeine, stock solutions, and test concentrations

The chemical compound used in the present study was CAF (1,3,7 trimethylxanthine), from Sigma Aldrich (CAS: 58-08-2; molecular weight 194.19 g/mol; 99% purity). The test solutions for the different bioassays were obtained from caffeine (CAF) stock solutions prepared by dilution in the respective culture medium of the *D. magna* and ASTM hard water (ASTM 1980). ASTM hard water refers to the standard synthetic medium recommended by the American Society for Testing and Materials (ASTM 1980), commonly used for culturing and testing aquatic organisms in ecotoxicological assays. It is prepared by dissolving calcium sulphate, magnesium sulphate, sodium bicarbonate, and potassium chloride in deionized water, resulting in a moderately hard water matrix with a total hardness of 160–180 mg/L as CaCO_3_ (ASTM 1980). For the *D. magna* assays, the stock solutions, nominal and actual (lowest and highest) concentrations tested, prepared for acute exposure, feeding inhibition assay, subchronic exposure, and chronic exposures, are summarized in Table 1. Caffeine concentrations for acute exposure were determined based on the literature data from acute toxicity (OECD 2012a; Rodrigues et al. 2025). CAF concentrations defined for the feeding inhibition assay were based on EC and LC values determined in the previous acute test as well as the biochemical results, considering equivalent exposure time. For sub-chronic and chronic exposures, concentrations were defined based on EC_5_ and EC_50_ determined for 48 h CAF exposure (Table 2).

**Table 1:** **–** Stock solutions (mg/L), range of nominal concentrations, and actual concentrations (mg/L) of caffeine (CAF) measured at the beginning of the bioassays with *D. magna*. * SE - standard error

Bioassays	Stock solution (mg/L)	Range of nominal [CAF] (mg/L)	Actual – lowest and highest [CAF] ± SE* (mg/L)
**Acute exposure (48 h)**	3500	27, 49, 88, 159, 286, 514, 926, 1668, and 3000	26 ± 4.5 and 2400 ± 50
**Feeding inhibition assay** (24 h)	1000	249, 296, 352, 419, 499, 593, 706, and 1000	220 ± 15 and 890 ± 36
**Subchronic exposure (10 d)**	120	3.8, 7.5, 15, 30, and 60	3.6 ± 0.5 and 59 ± 7.5
**Chronic exposure (21 d)**	100	4.6, 6.9, 10.4, 15.6, 23.3, and 35	4.5 ± 0.2 and 31 ± 4

**Table 2:** The number of normal/mobile (N), irregular swimming behavior (ISB), immobile (I), and dead (D) of *D. magna*, after 24 and 48 h of (acute) exposure to a range of caffeine concentrations (mg/L). E(L)C_50_ (95% confidence interval) for 24 and 48 h are also presented.

		24 h				48 h			
		N	ISB	I	D	N	ISB	I	D
**Caffeine** **(mg/L)**	**0**	40	0	0	0	40	0	0	0
	**27**	40	0	0	0	39	0	0	1
	**49**	37	3	0	0	26	13	0	1
	**88**	18	14	8	0	1	23	13	3
	**159**	13	10	17	0	0	0	35	5
	**286**	16	15	9	0	0	0	37	3
	**514**	4	22	14	0	0	0	33	7
	**926**	0	4	35	1	0	0	26	14
	**1668**	0	0	33	7	0	0	0	40
	**3000**	0	0	0	40	0	0	0	40
**mg/L**	**EC**_**5**_	25.23 (15.35–35.11)				33.74 (27.68–39.79)			
	**EC**_**50**_	130.8 (107–154.54)				53.06 (48.26–5787)			
	**LC**_**5**_	1368.2 (1183.1–1553.3)				149.35 (94.42–204.28)			
	**LC**_**50**_	1933.2 (1760.32–2106.07)				774.10 (632.03–916.17)			

LC_x_ values were estimated based on dead organisms, whereas EC_x_ values were calculated using the combined categorization of dead (D), immobile (I), and irregular swimming behavior (ISB) organisms, representing an integrated endpoint of acute effects. n_total_ = 40 organisms

### Test organism: Daphnia magna and culture maintenance

Cultures of *D. magna* were maintained in the laboratory for several generations through asexual reproduction (parthenogenesis), ensuring the same genetic variability. The cultures of *D. magna* were kept in a climate chamber with controlled conditions of temperature (20 ± 2 °C) and a photoperiod of 16 h of light and 8 h of darkness. *D. magna* was cultivated in a synthetic aqueous medium, ASTM “hard water” (ASTM 1980), supplemented with the standard organic additive, *Ascophyllum nodosum* extract (1:156) (Baird et al. 1989). The cultures were fed every 2 days with the green microalga *Raphidocelis subcapitata* (3.0 × 10^5^ cells/mL/day). The cultures were regularly renewed using neonates from the third to the fifth brood.

### Bioassays

#### Acute exposure (48 h)

The acute immobilization/mortality assay was performed according to the standard guideline 202 (OECD 2004), for 48 h of exposure, with some adaptations related to the evaluation of effects on swimming behavior (Diogo et al. 2023b). Each experiment consisted of eight replicates, each one with 5 organisms (less than 24 h old and born between the 3^rd^ and the 5^th^ brood). Exposures took place in glass vessels, each one with 30 mL of test concentration or control (Table 1). The assay was performed under controlled conditions similar to the maintenance of cultures (temperature of 20 ± 1 °C and a photoperiod of 16 h light:8 h dark). No feeding was provided during the 48 h exposure, as recommended by the guideline. In the acute assays, organisms were less than 24 h old, before the first molt, non-feeding, and relying exclusively on their internal nutrient reserves. The organisms were observed after 24 h and 48 h of exposure and were evaluated according to four behavioral categories, according to Diogo et al. (2023b): normal behavior (N); dead (D); immobilized (I); with irregular swimming behavior (ISB). Determination of LC_5_, LC_50_, EC_5,_ and EC_50_ values, and corresponding confidence intervals at 95%, were calculated for 24 h and 48 h of CAF exposure. After the exposure period (48 h), three replicates per treatment (with 6 or 7 organisms, randomly selected from surviving organisms after acute exposure) were prepared for posterior quantification of lipid peroxidation (TBARS levels), and another 3 replicates were used to quantify neurotoxicity (AChE activity). These biochemical markers were performed in triplicate for each assay to ensure the robustness and reproducibility of the results, while maintaining independent biological replicates for each biomarker.

#### Feeding Inhibition assay (24 h)

The feeding inhibition assay was conducted according to Rodrigues et al. (2022). The assay was conducted in 6-well microplates, with each plate corresponding to a concentration of CAF (Table 1). In each replicate, 12.5 mL of each CAF concentration was placed, along with *R. subcapitata* (3.0 × 10^5^ cells/mL/day). Subsequently, the initial absorbance (λ = 440 nm) of each replicate (well) was measured using a benchtop spectrophotometer (UV-1600PC spectrophotometer). After that, 5 neonates of *D. magna* (4 to 5 days old, born between the 3^rd^ and 5^th^ brood) were placed in each well of the microplate (i.e., 5 replicates, each with 5 organisms). In each microplate, one of the wells served as the control, containing only the concentration of CAF and *R. subcapitata* to account for the potential algal growth during the assay period. The assay was conducted for 24 h under controlled temperature conditions (20 ± 2 °C) and in darkness to ensure that there was no growth of the microalgae during the assay period. At the end of the exposure period, organisms from each CAF concentration were collected for further biochemical analyses (3 replicates with 5 organisms to evaluate oxidative stress biomarkers, and 3 replicates with 3 or 4 organisms to evaluate cholinergic neurotransmission biomarkers). After removal of the organisms, the final absorbance (λ = 440 nm) was read on a benchtop spectrophotometer (UV spectrophotometer 1600PC). Feeding rate results were expressed according to Allen et al. (1995).

#### Subchronic exposure (10d)

A simplified version of the standardized chronic assay for *D. magna* (OECD No. 211,) was conducted for a 10-day exposure (sub-chronic assay), according to the description in Diogo et al. (2023b). Previous studies have shown that this type of shorter exposure allows us to evaluate the effects of sublethal concentrations in terms of growth, energy allocation, and reproduction, as well as simulate the worst scenarios in terms of oxidative stress until the birth of the first brood (Guilhermino et al. 1999; Masteling et al. 2016; Diogo et al. 2023b). For each CAF concentration (Table 1), 21 replicates (one individualized organism per replicate) were performed, with 30 mL of ASTM, plus organic extract of *Ascophyllum nodosum* (1:156), and each one with one neonate of *D. magna*, less than 24 h old and born between the 3^rd^ and 5^th^ litter. The assay was performed under controlled conditions similar to the maintenance conditions. At the beginning of the assay, 21 neonates from the same brood as those used in the assay were measured using a binocular microscope with an attached camera (Leica MZ7.5) and with the help of Leica Application Suite (LAS) software to record the initial size. Every 48 h of exposure, the culture medium was completely renewed, and the organisms were fed daily. The assay was observed daily to record mortality, count and discard the neonates, and to account for the parameters related to the organisms’ life history: (i) age at first reproduction (number of days to the first brood), (ii) fecundity of the 1st brood (number of neonates produced by each female in the first brood – N1), (iii) somatic growth rate (increase in body length per day, day⁻¹), and (iv) population growth rate (*r*, assessment of population growth potential, day⁻¹), calculated according to the descriptions in Antunes et al. (2003). At the end of the exposure period, all surviving organisms were measured to record the final size and collected into Eppendorf microtubes (three replicates with 3 or 4 organisms from each treatment to evaluate oxidative stress and neurotoxicity biomarkers), then stored at -80 °C for subsequent biochemical analyses.

#### Chronic exposure (21d)

The chronic assay was conducted according to the standardized OECD No. 211 protocol (OECD, 2012b), for 21 days, at several CAF concentrations (Table 1). The entire procedure of this assay was similar to the sub-chronic assay (one individualized organism per replicate; 21 replicates per treatment). During the assay, parameters related to the organisms’ life history were assessed, such as (i) age at first reproduction; (ii) fecundity; (iii) reproductive output (the reproductive effort of each organism); (iv) the number of broods; (v) somatic growth rate; and (vi) population growth rate (Antunes et al. 2003). At the end of the exposure period, all surviving organisms were measured to record the final size, and subsequently collected for further biochemical analyses (*see* sub-chronic exposure).

### Determination of real CAF concentrations

For the quantification of CAF concentrations (Table 1), 2 samples of the lowest and highest concentration tested for each assay, at the beginning of the assay (0 h), were randomly collected (Table 1). It was only feasible to analyze the lowest and highest nominal concentrations for each assay. This approach aimed to indicate the potential difference between nominal and actual concentrations across the tested range and verify the stability of the CAF solutions. Samples were immediately frozen at -20 °C until further quantification of CAF. For this quantification, the samples were filtered using a regenerated cellulose syringe filter (0.22 mm pore). One millilitre of the filtered sample was spiked with 10 ng of internal standards of Caffeine-(trimethyl)-13C3 per aliquot of sample. The samples were analyzed by a DIONEX UltiMate 3000 ultra-high pressure liquid chromatography (UPLC) system (Thermo Scientific, Waltham, MA, USA) coupled to a triple quadrupole mass spectrometer (MS/MS) (TSQ QUANTIVA, Thermo SCIENTIFIC, Waltham, MA, USA). An Acquity UPLC BEH-C18 column (Waters, 100 mm × 2.1 i.d., 1.7 μm particle size from Waters Corporation, Manchester, UK) was used as an analytical column. The obtained data were evaluated using TraceFinderTM 3.3. software (Thermo Fisher).

The linearity of the calibration curve was tested in the range from 0.01 mg/L to 5000 mg/L. The limit of quantification (LOQ) was calculated as one-quarter of the lowest calibration point in the calibration curve where the relative standard deviation of the average response factor was < 30%. LOQ was 0.8 mg/L. The precision of the method was evaluated by the repeatability of the study. For this purpose, all samples were prepared in triplicate. No target compound was detected in method blanks and control samples.

### Biochemical markers

For the quantification of oxidative stress biomarkers, the samples were sonicated in 1 mL of phosphate buffer (50 mM, pH 7, with 0.1% Triton X-100) using an ultrasonic cell disruptor (Misonix Microson Ultrasonic Cell Disruptor XL). After homogenization, the samples were centrifuged at 14,000 rpm for 10 min at 4 °C, and the supernatant was divided into aliquots for subsequent quantification of CAT and GSTs activities, and TBARS levels. Quantifications were adapted for 96-well microplates and performed in quadruplicates, except for TBARS levels, which were measured in triplicate, according to Diogo et al. (2023a, b) and Rodrigues et al. (2021, 2022).

For the quantification of AChE activity, samples were homogenized in 500 µL of phosphate buffer (pH 7.2, at 4 °C) using an ultrasonic disruptor (Misonix Microson Ultrasonic Cell Disruptor XL). The samples were then centrifuged at 4 °C for 5 min at 6,000 rpm. AChE activity was determined according to Diogo et al. (2023a, b). The total protein concentration in all samples was determined following the methodology described by Bradford (1976), adapted for microplate use, to express the biochemical markers per mg of total protein.

### Statistical analysis

The estimation of LC_50_ values was performed with the results of dead organisms. In contrast, the estimation of EC_50_ values was performed with the sum of the results of the effects categorization of the dead (D), immobile (I), and irregular swimming behavior (ISB) binomial data [using the R package “drc”; (Ritz and Streibig 2005)] with a special case of the log-logistic dose-response model, where the asymptotes of the curve are fixed to be 1 (all organisms are dead, immobilized or with irregular swimming behavior) and 0 (none are immobile), following the rationale of Ritz (2010). To the food inhibition results of *D. magna*, the determination of EC_50_ values (using the delta method) and their respective 95% confidence intervals (CI_95%_) was performed by fitting a non-linear concentration-response toxicity model (LL3) using the drc package (Ritz and Streibig 2005) for the R software. Food inhibition was modelled as a continuous variable using a three-parameter logistic model, where the lower asymptotes of the curve were fixed at 0, as per Ritz (2010).

The results of feeding, life history parameters, and biochemical markers were checked for normality using the Shapiro-Wilk test and for homogeneity of variances using the Levene test. A one-way ANOVA was performed for all biomarker results, followed by Dunnett’s test (conducted to determine the differences between the CAF concentrations and the control group). All statistical analyses were conducted in SPSS Statistics v26, using a significance level of α = 0.05.

## Results

As shown in Table 1, the differences between nominal and measured concentrations for the lowest and highest treatments across all bioassays were minimal, with deviations generally within 5–10%. This consistency between nominal and actual concentrations indicates that the preparation of test solutions was reliable and stable throughout the experiments, supporting the validity of the ecotoxicological data. Given these small deviations, we are confident that the nominal concentrations accurately represent the exposure levels, even for the intermediate treatments that were not analytically verified.

### Acute exposure

#### Swimming behavior, immobilization, and mortality

The results of the swimming behavior, immobilization, and mortality of *D. magna* after 24 h and 48 h exposure to a range of CAF concentrations are shown in Table 2. Perturbations in swimming behavior (ISB) were observed in *D. magna*, even after 24 h of exposure, at ≥ 49 mg/L (Table 2). Regarding immobilization (I), *D. magna* experienced immobilization after 24 h of exposure to CAF at ≥ 88 mg/L (Table 2). Mortality (D) was also observed at concentrations from 926 mg/L after 24 h of exposure (Table 2). Extending the exposure duration to 48 h, the tested organisms displayed irregular swimming behavior (ISB) at concentrations of 49 and 88 mg/L (Table 2). Within the range of 88 to 926 mg/L, *D. magna* exhibited immobilization (I), while mortality (D) was recorded across all tested concentrations exceeding (≥ 27 mg/L), with 100% of mortality above 1668 mg/L, after 48 h. The E(L)C_5_ and E(L)C_50_ for 24 and 48 h are also presented in Table 2, indicating an increase in acute toxicity at 48 h, compared with the results of E(L)C_50_ for 24 h. These descriptive results suggest a trend towards increased toxicity over time.

**Fig. 1:**
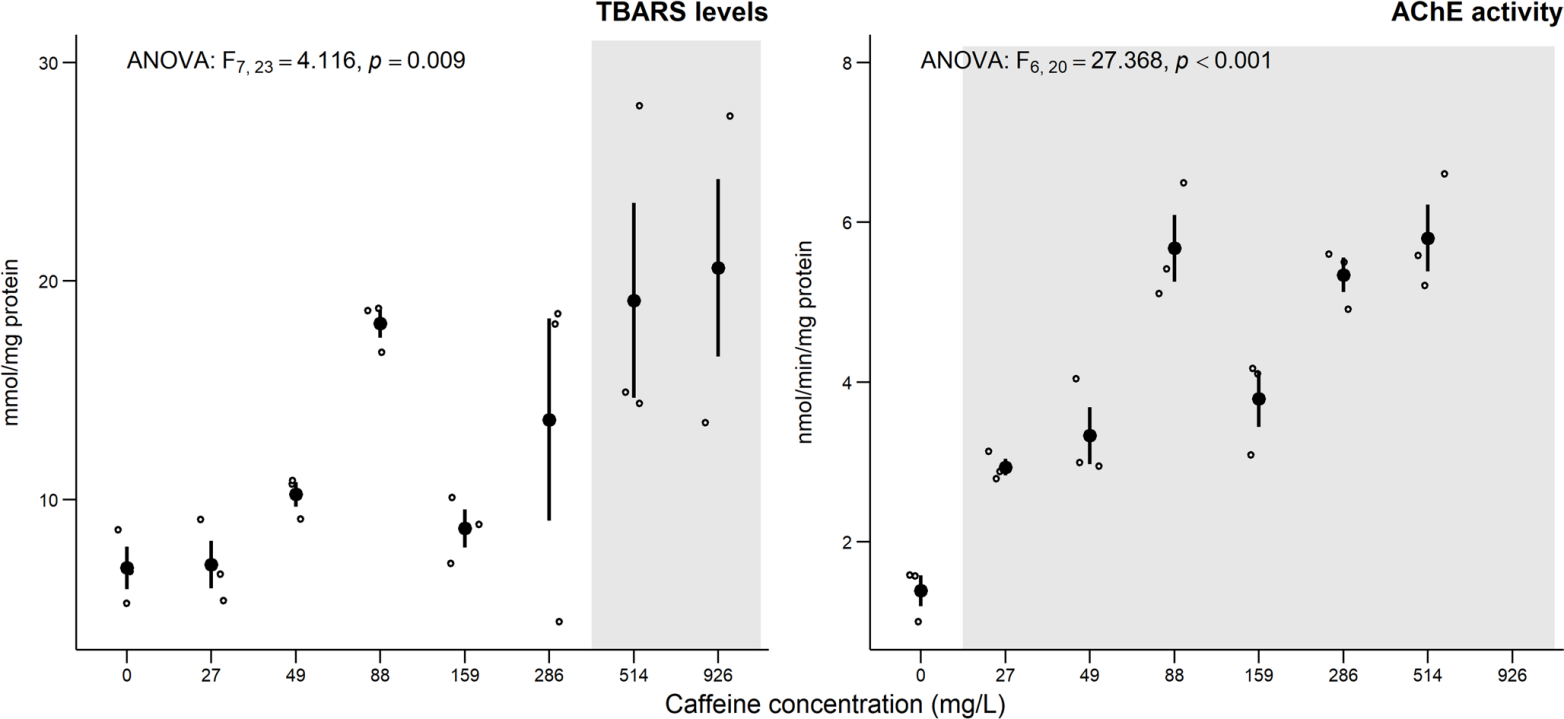
– Results of biochemical markers on *D. magna*, after acute exposure (48 h) to a range of CAF concentrations (mg/L). Data are expressed as mean (*n* = 3) ± standard error. ° stands for individual values (replicates). Grey shadows stand for significant differences compared to the control group (0 mg/L) (Dunnett’s test, *p* < 0.05)

The results of the biochemical markers of *D. magna* after 48 h exposure to CAF are presented in Fig. 1. After acute exposure to CAF, TBARS levels showed a significant increase at the two highest tested concentrations (514 and 926 mg/L) (Fig. 1). AChE activity also increased significantly at all tested concentrations (≥ 27 mg/L). The lack of results in biochemical markers (TBARS levels and AChE activity) content at the two highest concentrations (1668 and 3000 mg/L) is due to the absence of surviving organisms of *D. magna*.

### Feeding Inhibition assay

**Fig. 2:**
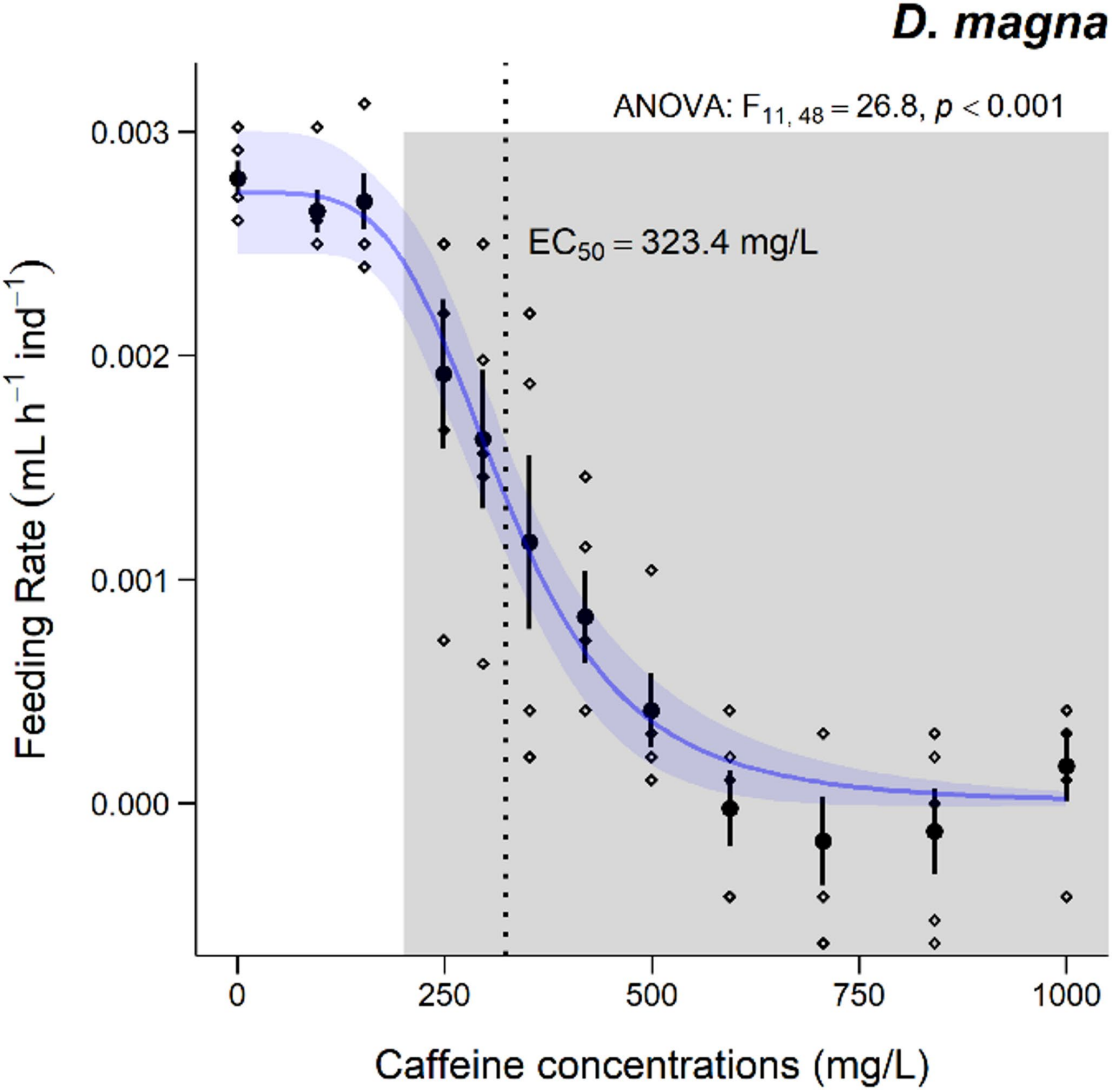
– Results for the feeding rate of *D. magna* after 24 h of exposure to a range of CAF concentrations (mg/L). Data are expressed as mean (*n* = 5) ± standard error. ° stands for individual values (replicates). Grey shadows stand for significant differences compared to the control group (0 mg/L) (Dunnett’s test, *p* < 0.05). EC_50_ value is also presented in the dotted line

The results of the feeding rate of *D. magna*, after 24 h exposure to different CAF concentrations, are shown in Fig. 2. As demonstrated, a significant decrease in the feeding rate was recorded at concentrations ≥ 296 mg/L of CAF. Regarding the feeding rate of *D. magna*, the EC_50_ (24 h) determined was 323.4 mg/L (CI_95%_: 289.15–358.74 mg/L).

**Fig. 3:**
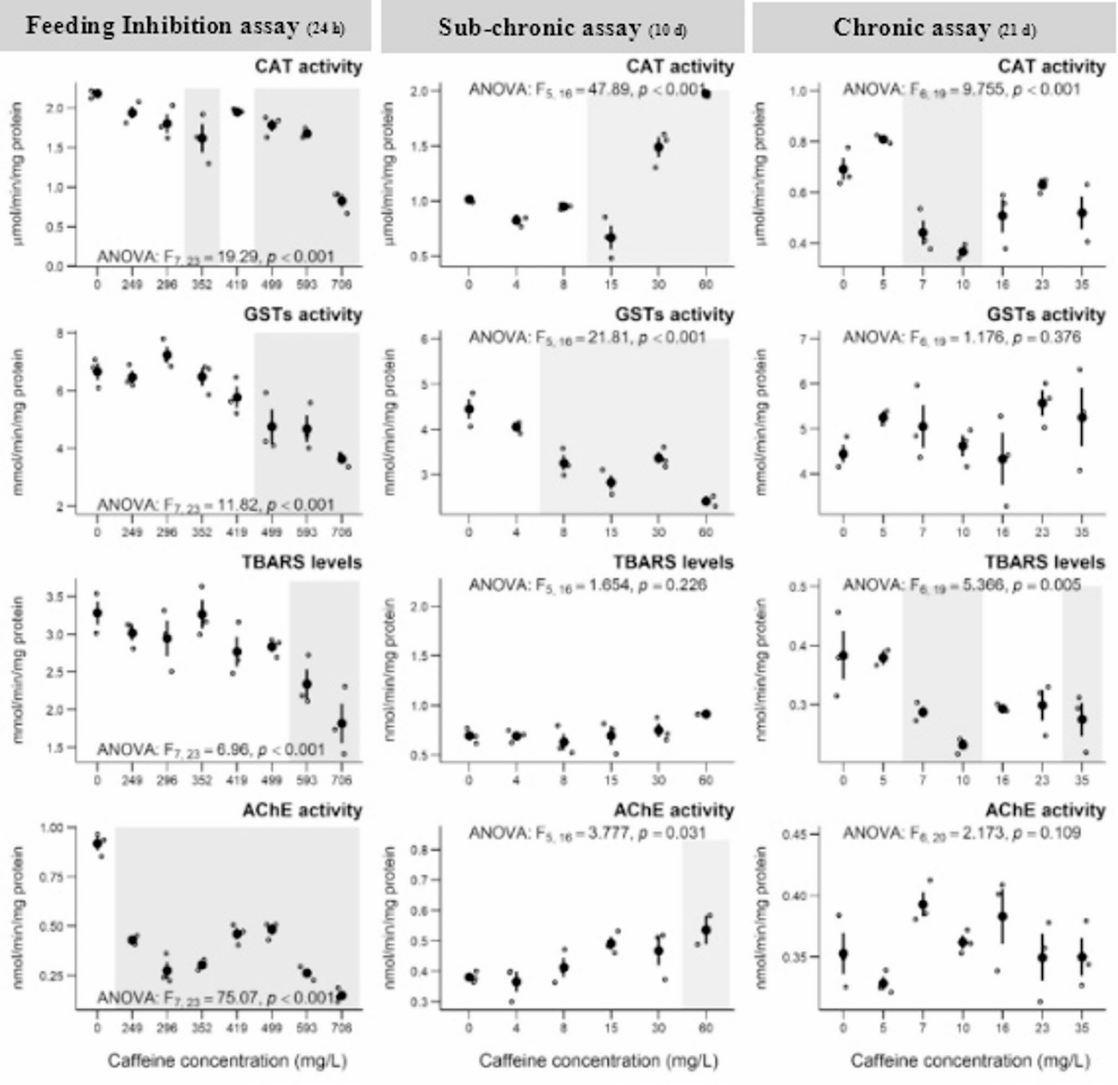
– Results of biochemical markers on *D. magna*, after 24 h (feeding inhibition assay), 10 d (sub-chronic assay), and 21 d (chronic assay) exposures to caffeine concentrations (mg/L). Data are expressed as mean (*n* = 3) ± standard error. ° stands for individual values (replicates). Grey shadows stand for significant differences compared to the control group (0 mg/L) (Dunnett’s test, *p* < 0.05)

The results of the biochemical markers of *D. magna* after 24 h exposure to CAF are presented in Fig. 3. Regarding CAT activity, a significant decrease was observed at concentrations of 352, 499, 593, and 706 mg of CAF/L. GSTs activity showed a significant decrease at the three highest concentrations (> 499 mg of CAF/L). TBARS levels also presented a significant decrease at the two highest concentrations (593 and 706 mg of CAF/L). Regarding AChE activity, a significant decrease occurred at all concentrations tested (Fig. 3).

### Sub-chronic and chronic exposures

The results of individual parameters (life history) of *D. magna* after subchronic exposure showed no significant differences for the age at first reproduction (Table 3). A significant increase in the fecundity of the first brood was observed in individuals exposed to 15 mg/L of CAF (Table 3). Regarding the somatic growth rate and rate of population increase, a significant decrease was observed after 10 days of exposure (Table 3), but only at the highest concentration tested (60 mg/L). The results of the biochemical markers quantified in *D. magna* after subchronic exposure (10 days) to CAF are presented in Fig. 3. A significant decrease in CAT activity was recorded at the concentration of 15 mg/L, while a significant increase at the higher concentrations tested was observed (30 and 60 mg/L) (Fig. 3). Regarding GSTs activity, a significant decrease occurred at all concentrations ≥ 8 mg/L. For TBARS levels, no significant changes were observed after 10 days of exposure (Fig. 3). Concerning AChE activity, a significant increase was only observed at the highest concentration tested (60 mg/L of CAF).

**Table 3:** **–** Results of one-way ANOVA for subchronic (10 days) and chronic (21 days) exposures of *D. magna* to caffeine, showing degrees of freedom (d.f.), F-values, and *p*-values for the evaluated life-history parameters.

Exposure	Parameters	d.f.	F	*p*
**Subchronic** **(10 d)**	Age at first reproduction	5, 95	2.083	0.075
	Fecundity of the first brood	5, 95	3.035	**0.014**
	Somatic growth rate	5, 106	6.688	**< 0.001**
	Rate of population increase	5, 125	8.498	**< 0.001**
**Chronic** **(21 d)**	Age at first reproduction	6, 142	1.88	0.088
	Fecundity of the first brood	6, 145	1.48	0.189
	Somatic growth rate	6, 133	1.02	0.413
	Rate of population increase	6, 146	0.791	0.578
	Number of broods	6, 134	2.43	**0.029**

Bold values stand for significant differences (*p* < 0.05).

Regarding the chronic exposure (21 days of exposure to CAF), no significant effects were observed on the individual parameters (life history) of *D. magna*, such as age at first reproduction, reproductive output, somatic growth rate, and rate of population increase (Table 3) under the tested conditions. However, a significant decrease was observed in the number of broods produced at the concentration of 10 mg/L (Table 3). Fecundity was also significantly affected, with a significant decrease (Table 3) at concentrations of 10, 16, and 35 mg/L. After chronic exposure, a significant decrease in CAT activity was observed at concentrations of 7 and 10 mg/L (Fig. 3). However, no significant differences were observed in GSTs and AChE activities (Fig. 3). TBARS levels showed a significant decrease at concentrations of 7, 10, and 35 mg/L (Fig. 3).

## Discussion

### Acute exposure

#### Swimming behavior, immobilization, mortality, and biochemical markers

The results of behavioral responses of *D. magna* following acute exposure to CAF demonstrated a clear dose-response toxicity trend. Analysis of EC_5_ and EC_50_ values indicates that relatively low concentrations of CAF already impact the mobility of *Daphnia*. It was observed that the EC_50_ for 24 h was 130.8 mg/L, while for 48 h, this value was reduced to 53.06 mg/L. This phenomenon was also observed in the LC_50_ values, where lethality increased considerably between the exposure times, going from 1933.2 mg/L in 24 h to 774.1 mg/L in 48 h. Although the 95% confidence intervals partially overlap between 24 h and 48 h, the marked reduction in the EC_50_ and LC_50_ central values supports a time-dependent increase in CAF toxicity. This pattern is consistent with cumulative effects reported in the literature for *Daphnia* under similar exposures (e.g., Pashaei et al. 2023). Overall, it was evident that the presence of CAF in the environment influenced both swimming behavior and mortality in *D. magna*. Mortality was initially recorded after 24 h only at the highest concentrations tested (> 926 mg/L). However, after 48 h, mortality was observed at all tested concentrations, and from 1668 mg/L, the mortality rate reached 100%, suggesting a critical threshold of toxicity, alongside a reduction in EC_50_ values. Martinez Gomez et al. (2015) also demonstrated acute toxicity of CAF in the rotifer *Pationus patulus* at two concentrations tested (450 and 525 mg/L) after 48 h of exposure, with a mortality of 50% at the highest concentration reported.

Notably, the onset of behavioral changes occurred well below lethal thresholds, reinforcing the ecological relevance of sublethal endpoints in ecotoxicological studies. Behavioral disruptions in *D. magna* may compromise their ability to feed, evade predators, and reproduce, ultimately affecting population dynamics and aquatic food web stability (Barata et al. 2001; Nørum et al. 2010). As such, CAF concentrations detected in aquatic environments, particularly those influenced by wastewater effluents, could pose a risk to non-target organisms, even at levels insufficient to cause direct mortality. Overall, these results highlight the importance of incorporating behavioral endpoints into standard acute toxicity assessments. Additional studies are needed to better understand the mechanisms by which CAF affects the physiology of *D. magna*, as well as to assess possible impacts at ecosystem levels. Furthermore, investigation of environmentally relevant concentrations would allow for a more accurate estimation of the ecological risk of CAF in natural aquatic environments.

TBARS levels results suggests that CAF can generate reactive oxygen species (ROS) that exceed the antioxidant capacity of *D. magna*, resulting in oxidative damage (lipid peroxidation) (Rodrigues et al. 2021), demonstrated as significant increase at the two highest concentrations tested (514 and 926 mg/L). This study also demonstrated that AChE activity increased significantly at all concentrations tested (27–926 mg/L), indicating a possible influence of CAF on cholinergic neurotransmission (Muñoz-Peñuela et al. 2022) and reflects an initial stimulatory or compensatory response to CAF-induced neuroexcitation, as reported in invertebrates exposed to neuroactive compounds (Aguirre‑Martínez et al., 2016; Vieira et al. 2022). Previous studies suggest that psychoactive substances, such as CAF, can modulate AChE activity in aquatic organisms, which may be related to cellular defence mechanisms or even direct adverse effects on the nervous system (Barata et al. 2005; Ruiz-Oliveira et al. 2019; Munoz-Peñuela et al. 2022; Remley et al. 2023). However, studies with CAF for this type of organism and exposure time are scarce, so more future studies are needed and these should consider complementary evaluations, including behavioral biomarkers and gene expression of antioxidant enzymes, for a more comprehensive understanding of the mechanisms underlying the effects of caffeine in aquatic organisms.

### Feeding Inhibition assay

#### Feeding rate

In this study, it was demonstrated that the feeding performance of *D. magna* was affected by CAF exposure at concentrations equal to or higher than 296 mg/L. It can be observed that there is a dose-response relationship, meaning that the higher the caffeine concentration, the lower the feeding rate observed in *D. magna*. Lu et al. (2013) also noted that the feeding rate of *D. magna* was significantly reduced after 5 h of exposure, even at lower concentrations of CAF (> 0.12–10 mg/L), and at the highest concentration (10 mg/L), the feeding rate was inhibited by about 91%. Lu et al. (2013) reported that CAF affects the nervous system by inhibiting AChE activity, which disrupts cholinergic neurotransmission (Farias et al. 2021), causing a loss of coordination and/or paralysis, altering feeding behaviour, and leading to a reduction in absorption/filtration rates. Thus, changes in the feeding rate of *D. magna* due to exposure to emerging contaminants like CAF may have implications for the aquatic ecosystem. Barata et al. (2005) demonstrated that exposure to certain contaminants of emerging concern (CEC; e.g., endosulfan, cadmium, menadione) can induce changes in feeding behaviour, as observed in the feeding inhibition assay of the present study. The reduction in the feeding rate of *D. magna* may lead to decreased intake of essential nutrients, resulting in reduced growth and reproduction. Furthermore, this could negatively impact ecological balance, as *D. magna* plays a key role in controlling phytoplankton in aquatic ecosystems, contributing to good water quality. Maintaining the health and population viability of *D. magna* is also essential for ecological stability, as they serve as food for predators (secondary consumers such as fish).

#### Biochemical markers

Free radicals, particularly ROS, are unstable molecules that can cause significant oxidative stress and damage to proteins, lipids, and DNA (Diogo et al. 2023a, b). Organisms have antioxidant defences, including enzymes such as CAT and GSTs, to combat these negative effects, maintaining the redox balance essential for cellular health in organisms (Rodrigues et al. 2016).

CAT is responsible for the removal of hydrogen peroxide (H_2_O_2_) from the cell (Muñoz-Peñuela et al. 2022; Rodrigues et al. 2022), and the decrease in CAT activity in organisms exposed to CAF indicates oxidative stress. As reported in other studies, CAF can induce an increase in ROS production, promoting a burden on the antioxidant defence system of *D. magna* (Muñoz-Peñuela et al. 2022), leading to inhibition or direct damage to antioxidant enzymes (Aguirre-Martínez et al. 2013), such as CAT. High concentrations of CAF may reduce the ability of CAT to neutralize hydrogen peroxide, decreasing its activity. Other studies have already shown that exposure to CAF activates antioxidant defences (SOD, CAT, GSTs) in aquatic organisms [e.g., *Danio rerio* (0.16–50 µg CAF/L; 28 days; Diogo et al. 2023a); *Ruditapes philippinarum* (0.5, 3, and 18 µg CAF/L; 28 days; Cruz et al. 2016)], indicating that CAF promotes oxidative stress (Li et al. 2020; Diogo et al. 2023a). However, the concentrations tested in the aforementioned studies are lower than those tested in the present work; nonetheless, both studies demonstrate toxicity.

Since GSTs are a group of isoenzymes involved in phase II metabolism (i.e., they catalyse the conjugation of various metabolites or toxic compounds with glutathione - GSH) (Rodrigues et al. 2021, 2022), the decrease in their activity limits the elimination of ROS and electrophilic compounds, which can induce cellular damage and compromise cellular integrity and function (Diogo et al. 2023a). This decrease in GSTs activity may be associated with several factors: (1) an increase in oxidative stress, meaning that CAF may induce excessive ROS production, causing oxidative damage or inactivation of GSTs (Aguirre-Martínez et al. 2013); (2) direct damage to the enzyme, i.e., CAF may induce structural modifications in proteins, including GSTs, through processes such as oxidation or nitrosylation, reducing their affinity for substrates or decreasing their catalytic efficiency (Daniel et al. 2019); (3) interference in the synthesis or regeneration of glutathione, meaning that CAF may interfere with the synthesis or regeneration of GSH, leading to a lower availability of this essential cofactor, and with less GSH available, GSTs activity may decrease (Diogo et al. 2023b); (4) GSH is a non-enzymatic antioxidant defence, and on its own, it can combat ROS (Rodrigues et al. 2016), becoming less available for conjugation *via* GSTs; (5) negative regulation of GSTs gene expression, meaning that exposure to CAF may lead to negative regulation of the expression of genes encoding GSTs, resulting in lower production of GSTs and, consequently, a decrease in their enzymatic activity (Nunes et al. 2018); (6) direct inhibition of enzymatic activity, as CAF may act as a competitive or allosteric inhibitor of GSTs, and if CAF or some by-product binds to the active or regulatory sites of GSTs, it may reduce the enzyme’s efficiency in catalysing the conjugation of GSH with toxic substrates (Nunes et al. 2018).

The significant decrease in TBARS levels may indicate that detoxification and antioxidant defence pathways (e.g., CAT and GSTs activity, or others not quantified) were effective in combating the excess of free radicals, preventing lipid peroxidation (Diogo et al. 2023b). Contrary to what was observed in this study, other works, particularly with estuarine and marine organisms (e.g., *Ruditapes philippinarum*, *Hediste diversicolor*, *Arenicola marina*), demonstrated that the antioxidant defence was not able to neutralize free radicals, and consequently, LPO was observed (Aguirre-Martínez et al. 2016; Pires et al. 2016; Li et al. 2020; Vieira et al. 2022). Santos-Silva et al. (2018) reported that 0.3, 3, and 30 µg/L of CAF caused an increase in TBARS levels in the liver and brain of the fish *Prochilodus lineatus* after 24 h of exposure.

Our findings with *D. magna* showed no significant changes in CAT, GST, or TBARS levels even at much higher CAF concentrations (up to 296 mg/L for CAT, 419 mg/L for GSTs, and 499 mg/L for TBARS). The absence of changes in oxidative homeostasis in *D. magna* even at high CAF concentrations, can be associated with species-specific differences in sensitivity and metabolism, variations in time and conditions of exposure, distinct pathways and levels of CAF accumulation, possible hormetic effects or enzyme inhibition at low concentrations, and methodological differences in enzyme measurement, compared with previous studies (Aguirre-Martínez et al. 2016; Cruz et al. 2016; Pires et al. 2016; Li et al. 2020; Vieira et al. 2022; Diogo et al. 2023a). In general, the lack of antioxidant modulation in *D. magna* at high CAF concentrations highlights the importance of considering multiple biological models and endpoints to assess environmental risks of contaminants like CAF.

Proper AChE activity ensures balance in nerve signalling, allowing electrical impulses to be efficiently regulated in the nervous and muscular systems of *D. magna*. In this study, *D. magna* exposed to high concentrations such as 248 to 716 mg/L of CAF demonstrated significant inhibition of AChE activity, which may reflect enzyme inhibition or metabolic downregulation due to stress, resource reallocation, and age‑related differences in sensitivity, compared with organisms acutely exposed (*see* Section - Swimming behavior, immobilization, mortality, and biochemical markers). These results corroborate another study where AChE activity decreased after exposure to increasing concentrations of CAF in *Danio rerio* (≥ 0.0088 mg/L, 7 days; Farias et al. 2021), demonstrating disruptions in cholinergic neurotransmission. Interestingly, the concentrations at which significant alterations in feeding rate were observed (≥ 249 mg/L) coincide with cholinergic disruptions mediated by AChE. Although no formal correlation analysis was performed, this overlap between biochemical and behavioral responses supports the mechanistic link between neurotoxicity and altered food performance. Cholinesterase inhibition has often been associated with feeding inhibition, as neurotoxic effects interfere with the organism’s feeding ability (Rodrigues et al. 2021). For example, CAF may cause a blockade of adenosine receptors, preventing adenosine from binding to its receptor (Remley et al. 2023). This blockade may indirectly affect the release of neurotransmitters (e.g., dopamine, gamma-aminobutyric acid (GABA), and glutamate), causing neurotransmission inhibition, and thus affecting the behavior of organisms (Ruiz-Oliveira, 2019), including not only changes in feeding but also in escape response and reproduction (Barata et al. 2005; Munoz-Peñuela et al. 2022).

### Subchronic and chronic exposure

#### Life history parameters

CAF is considered a low-risk CEC for aquatic biota, as the concentrations detected in various aquatic matrices are generally very low (ng to µg/L), remaining below the levels considered dangerous for most aquatic species (Marcantonio et al. 2023). However, its increasing consumption and detection in aquatic matrices over the years, combined with the mixture of other compounds in ecosystems, and its bioaccumulation potential in some aquatic species [e.g., *Perna viridis*, 1.55 ng/g (Bayen et al. 2015); *Gerres oyena*, 73.6 ng/g (Ali et al. 2018); *Corbicula fluminea*, 7.8 ng/g (Burket et al. 2019)] highlight the need for detailed and continuous analysis of its potential environmental risk (Dafouz et al. 2018). However, few studies have evaluated the effects and impacts of stimulants (particularly CAF) on aquatic organisms, especially on crustaceans, particularly at the behavioral (e.g., reproductive), physiological, and metabolic levels (Nunes et al. 2022). After subchronic and chronic exposure, our results showed that different concentrations of CAF did not affect the age at first reproduction of *D. magna*. However, Lu et al. (2013) demonstrated that the age at first reproduction of *D. magna* was significantly delayed after prolonged exposure (21 days) to CAF (0.12, 0.37, 1.11, 3.33, and 10 mg/L) at lower concentrations compared to those tested in the present study, highlighting potential differences in organismal age, exposure duration and conditions, tested concentrations and physiological state of organisms.

The fact that fecundity increased in the subchronic assay may be associated with an initial adaptive response, that is, a compensatory (“overcompensation”) response to stress caused by the toxicant (Lu et al. 2013). This type of response may occur as an attempt to ensure the survival of the species (Martins 2013). However, after chronic (more prolonged) exposure, the toxic effects of CAF may accumulate in *D. magna*, leading to a decrease in fecundity. The results of the chronic assay align with those of Lu et al. (2013), as they also demonstrated that at concentrations of 0.12 to 10 mg/L of CAF, a significant decrease in fecundity occurred.

In contrast, Lu et al. (2013) showed that after exposure of *D. magna* for 21 days (0.12–10 mg/L), a significant decrease in reproductive output at all tested concentrations was observed. The fact that no significant differences in reproductive output were observed after subchronic exposure to CAF may be due to several factors: (1) acclimation - the exposed organisms may have developed adaptation mechanisms to the stress induced by CAF over time, managing to adjust their physiological processes to mitigate the negative effects, thereby maintaining their reproductive output (De Felice et al. 2020); (2) energy reserves and resource allocation - *D. magna* may have been able to sustain reproduction by efficiently allocating its energy resources, even under stress, to reproduction as a means of ensuring species survival (Baird et al. 1989); (3) biological compensation – in response to environmental stress, some species tend to compensate by increasing early reproduction or maintaining reproduction over time, to ensure species survival (Li et al. 2020). Although chronic exposure to CAF did not affect the reproductive output of *D. magna*, the total number of broods produced per female significantly decreased at a concentration of 10 mg/L (3.7 ± 0.12 broods), compared to the control treatment (3.95 ± 0.05 broods).

The results of subchronic exposure corroborate those obtained by Lu et al. (2013), who observed a significant decrease in somatic growth rate at all tested concentrations (0.12–10 mg/L) after 21 days of exposure. Martins (2013) argues that metabolic stress due to the energy expenditure of organisms in response to stress (e.g., CAF) reduces the energy available for growth and development processes. On the other hand, the same authors indicate that interferences in the growth cycle may be associated with exposure to CECs (e.g., CAF), due to interference in hormonal regulation or cellular processes that are fundamental for growth (e.g., protein synthesis and cell division), resulting in slower growth or even growth inhibition (Martins 2013). Ruiz-Oliveira et al. (2019) argues that effects on the nervous system block adenosine receptors, which may lead to increased release of neurotransmitters such as dopamine and norepinephrine, and in *D. magna*, this nervous modulation can lead to changes in its behaviour and metabolism, diverting resources that would normally be allocated to somatic growth and other processes, such as stress responses. In this case, CAF, by altering the feeding behaviour of *D. magna*, may promote inefficient ingestion of food and essential nutrients, resulting in less energy available for growth, which may explain the reduction in the somatic growth rate.

Lu et al. (2013) observed a significant decrease in the rate of population increase of *D. magna* when exposed for 21 days to concentrations of 0.12 to 10 mg/L. This decrease may be directly related to the reduction in the fecundity of the organisms and indirectly related to the decrease in somatic growth rate (Braga 2017). Martins (2023) argued that smaller body size is associated with reduced reproductive capacity. In other words, organisms with lower somatic growth often reach sexual maturity later or remain smaller in size, which may lead to lower offspring production. On the other hand, there may have been a dispersion of energy towards survival; that is, exposure of *D. magna* to CAF may limit the energy that would normally be used for growth and reproduction, reallocating it to survival mechanisms, cellular repair, and stress response, which means less energy is available for offspring production (Braga 2017). Rodrigues et al. (2010) also reported that if exposure to CAF interferes with the growth of *D. magna*, the produced eggs may be of inferior quality, resulting in less viable neonates or those with lower survival rates, contributing to a decrease in the population growth rate, as fewer offspring reach adulthood to complete the reproductive cycle. Thus, exposure to CAF impairs the reproductive capacity of *D. magna*, which may be associated with oxidative stress, cellular dysfunction, hormonal disturbances (Martins 2013), or potential changes in feeding behaviour, as observed in the present study.

#### Biochemical markers

Muñoz-Peñuela et al. (2022) observed a decrease in CAT activity in the fish *Astyanax altiparanae* after 96 h of exposure to 27.5 µg CAF/L. In contrast to the chronic exposure results of the present study, Cruz et al. (2016) observed a significant increase in CAT activity at 3.0 and 18 µg CAF/L in *Ruditapes philippinarum* after 28 days of exposure. The results presented here reflect that CAF may have induced an increase in ROS production in the cells of *D. magna*, potentially exceeding the capacity of CAT activity, leading to a temporary inhibition or decrease in the activity of this enzyme. This process may occur because the excess ROS reduces the capacity of CAT activity or other cellular components, hindering their function (Muñoz-Peñuela et al. 2022). On the other hand, when there is an increase in CAF concentration, the cells of *D. magna* may activate adaptive antioxidant defence mechanisms, resulting in the induction of greater synthesis of CAT or the activation of inactive CAT, leading to an increase in catalytic activity (Aguirre-Martínez et al. 2013). The increase in CAT activity may represent an attempt to compensate for oxidative stress caused by the higher concentration of CAF, helping to protect the cells from lipid peroxidation (Aguirre-Martínez et al. 2013).

Several authors have demonstrated an increase in GSTs activity in response to CAF exposure in various aquatic organisms [e.g., *Corbicula fluminea* (Aguirre-Martínez et al. 2016), *Ruditapes philippinarum* (Cruz et al. 2016)]. However, according to the results obtained in the subchronic exposure, there was a significant decrease in GSTs activity starting from a concentration of 8 mg/L. The decrease in GSTs activity may result from the depletion of glutathione levels, inhibition of the enzymes, or cellular damage that affects the expression or functioning of these enzymes (Aguirre-Martínez et al. 2013). However, after chronic exposure, no significant changes were recorded in the activity of these antioxidant enzymes. This response may result from the adaptation of *D. magna* to CAF exposure, maintaining GSTs activity constant even under continuous stress (Nunes et al. 2018). Other authors emphasized that after prolonged exposure, the organism may have developed resistance or tolerance to the substance, demonstrating that GSTs activity did not need to be adjusted in response to continuous exposure (Nunes et al. 2018), which may have occurred after chronic exposure. Diogo et al. (2023a) also observed that after chronic exposure (28 days) to concentrations of 0.16 to 50.00 µg CAF/L, *Danio rerio* did not show significant changes in GSTs activity. In contrast to the results of the present study, Aguirre-Martínez et al. (2016) observed that the activity of this enzyme increased in *R. philippinarum* in response to CAF exposure (0.1, 5, 15, and 50 µg/L) after 14 days of exposure. Santos-Silva et al. (2018) also observed an increase in GSTs activity in *Prochilodus lineatus* after 7 days of exposure to CAF (0.3, 3, and 30 µg/L). According to Aguirre-Martínez et al. (2016) and Cruz et al. (2016), this increase occurs in response to oxidative stress caused by CAF exposure, activating GSTs to protect the organism, facilitating detoxification and elimination of toxic products, and protecting cells against oxidative damage.

Diogo et al. (2023a) reported that TBARS levels in *D. rerio* did not undergo significant changes after 28 days of exposure to CAF (0.16 and 50.00 µg/L). On the other hand, Santos-Silva et al. (2018) reported that 0.3, 3, and 30 µg/L of CAF induced an increase in TBARS levels in the liver and brain of *P. lineatus* after 7 days of exposure, reinforcing that CAF has the potential to induce lipid peroxidation under certain conditions and in aquatic organisms. However, under the conditions tested here, no oxidative damage was observed.

The results of AChE activity in the present study suggest that *D. magna* exhibited a potential adaptive response to the neurotoxic stress induced by CAF. Specifically, the observed increase and/or stabilization of AChE activity suggests that *D. magna* might compensate for potential disruptions in nerve transmission induced by CAF (Muñoz-Peñuela et al. 2022) during longer exposure periods. This increase during subchronic exposure could also indicate that CAF causes a neurotoxic effect, prompting the organism to produce more AChE to maintain neuromuscular homeostasis (Aguirre-Martínez et al. 2016). Contrary to the results presented here, several authors have demonstrated AChE activity inhibition after exposure to increasing concentrations of CAF (Aguirre-Martínez et al. 2016; Farias et al. 2021; Diogo et al. 2023a). Farias et al. (2021) observed that AChE activity decreased in zebrafish embryos exposed to a range of CAF concentrations (0.0088–50 mg/L) over 7 days. Diogo et al. (2023a) observed a decrease in AChE activity in *D. rerio* after chronic exposure (28 days) to 19.23 and 50 µg CAF/L. Aguirre-Martínez et al. (2016) evaluated the neurotoxicity of different CAF concentrations (5, 15, and 50 µg/L) in *R. philippinarum* and, after 14 days of exposure, concluded that the highest concentration tested (50 µg/L) significantly inhibited AChE activity, demonstrating neurotoxicity. Thus, changes in AChE activity serve as an important indicator of the effects of CAF exposure in the aquatic environment, reflecting a possible impact on the health and survival of *D. magna* populations (Diogo et al. 2023b).

### Proposal for ecotoxicological classification for CAF

Our study tested a wider range of CAF concentrations than those typically used in chronic sublethal studies or environmentally relevant. This was done intentionally to establish a comprehensive toxicity profile for *D. magna*, covering both environmentally relevant (e.g., extreme scenarios of environmental contamination) and higher, ecotoxicologically significant concentrations (i.e., induction of biological responses at different levels of biological organization) (Table 4). Testing concentrations well beyond environmentally detected levels is a common and recommended practice in ecotoxicological risk assessment (OECD, 2012b ), as it enables the identification of thresholds for multiple biological endpoints (e.g., antioxidant responses, AChE activity, feeding inhibition, reproductive performance). Therefore, although our upper concentrations exceed typical environmental levels, it integrates responses across a wide concentration gradient, improving its applicability for hazard characterization and comparative ecotoxicological assessments. According to the species sensitivity distribution plots present in Rodrigues et al. (2025), the hazardous concentration for 5% of freshwater species was 4.42 mg/L, which is included in the range of concentrations tested in the subchronic and chronic tests.

**Table 4:**
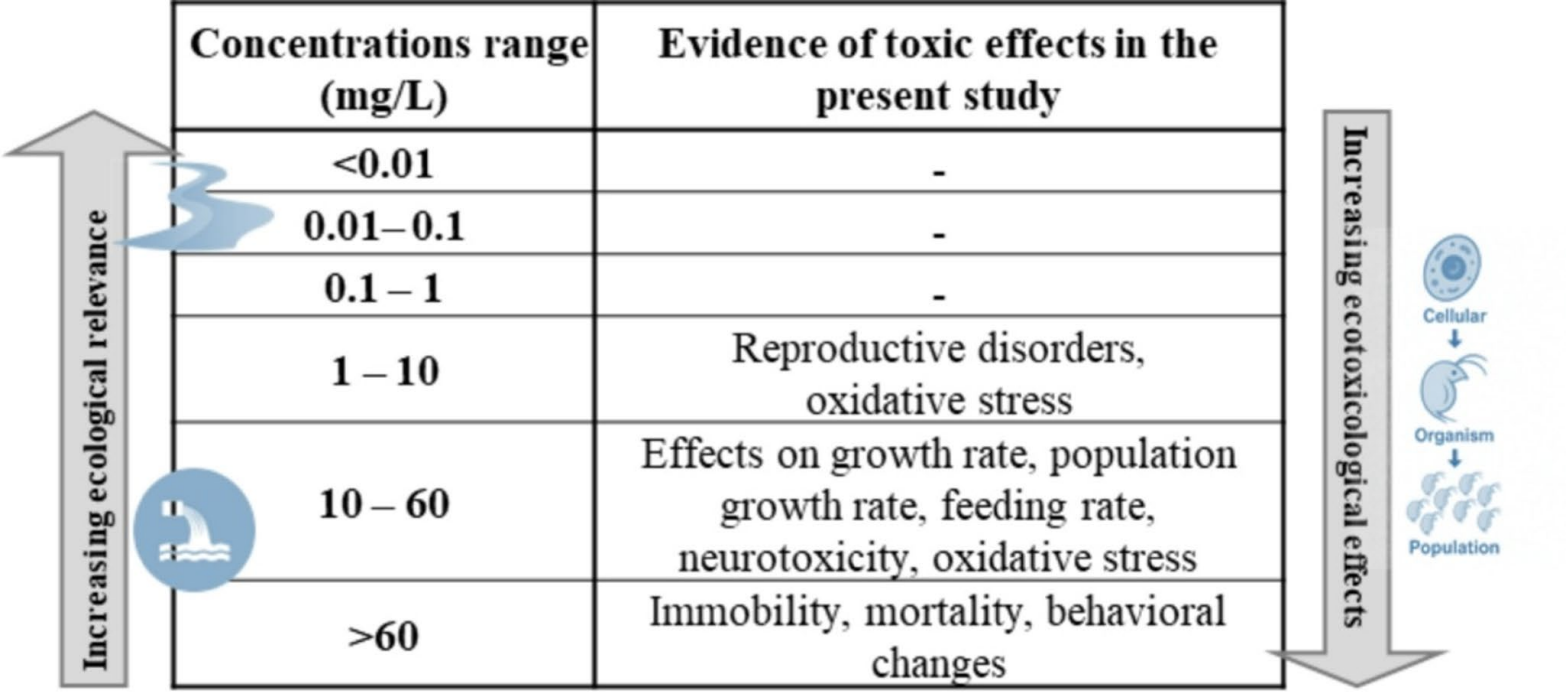
**–** Ecotoxicological results based on caffeine concentrations (CAF).

corresponds to concentrations of wastewater treatment plants (WWTP) effluents, and  corresponds to the concentrations of surface waters (rivers, lakes, and reservoirs) of CAF, according to the scientific literature.

Based on the biological responses presented here (individual and sub-individual; including acute, subchronic, chronic, and biochemical effects) and comparing them with environmental CAF concentrations, reported in the literature and concentrations tested, we defined an integrative toxicity summary for CAF (Table 4). The side arrows illustrate an inverse relationship between concentrations and ecotoxicological impact: as concentration increases, ecotoxicological impacts become more severe, while ecological and environmental risk assessments tend to indicate lower risk (Table 4). This approach contributes to the assessment of the ecological risk of CAF in aquatic environments and can assist in communicating levels of environmental concern, especially in locations where effluent treatment levels are ineffective.

Considering that the maximum environmental concentrations reported (wastewater effluents up to 86 mg/L; Nodler et al. 2010; Oliveira et al. 2015) approach or exceed thresholds for acute, subchronic and biochemical toxicity, such as reduced growth (60 mg/L), swimming behavior perturbations (≥ 49 mg/L), alterations in AChE activity (≥ 27 mg/L) and chronic reproductive effects (≥ 7 mg/L), CAF may be classified as having low inherent toxicity but remains a substance of potential concern. While it is generally regarded as posing low environmental risk, this classification may underestimate actual ecological threats, particularly in ecosystems exposed to untreated effluents or where removal efficiency is limited. Although acute toxicity occurs at higher concentrations, significant sublethal effects can precede mortality, potentially destabilizing populations and food webs. Therefore, the persistent presence of CAF in aquatic environments, along with its documented effects across various levels of biological organization (behavioral, physiological, and population), underscores the need for continued monitoring and regulatory attention. Finally, the principal limitation of this study should be acknowledged. The experiments were performed under controlled laboratory conditions, which do not fully replicate the complexity of natural ecosystems, where CAF often co‑occurs with other contaminants and environmental variables. These limitations should be considered when extrapolating our findings to real‑world scenarios and highlight directions for future research, and underscore the need for future research at environmentally relevant concentrations, including multi-stressor scenarios and integrative analyses, to better assess the ecological implications of caffeine exposure in aquatic environments.

## Conclusions

This study evaluated the potential ecotoxicological effects of CAF on *D. magna*, as there are few ecotoxicological studies involving non-target species, especially freshwater species. This study demonstrated that CAF induces significant sublethal and chronic effects on *D. magna*, affecting behavior, growth, reproduction, and key biochemical pathways. Notably, alterations in AChE activity and antioxidant defenses occurred at environmentally relevant concentrations, particularly in ecosystems exposed to untreated effluents or where removal efficiency is limited, reinforcing the ecological risk posed by this contaminant of emerging concern. Furthermore, CAF is still not listed as a substance to monitor in water bodies by the WFD, despite the observed increase in concentrations in the environment over the years and existing studies demonstrating its potential ecotoxicity. *D. magna* showed sensitivity to CAF exposure, both in short exposure periods (acute assay and feeding inhibition assay) and in long exposure periods (subchronic and chronic assays). These findings provide novel insight into the sensitivity of freshwater invertebrates to CAF and support its inclusion in future environmental monitoring frameworks. Considering the maximum environmental concentrations reported and all biological responses observed in this study, CAF may be classified as having low inherent toxicity, but remains a substance of potential concern. Given the widespread presence of CAF as a contaminant of emerging concern, further studies should explore chronic effects, potential mechanisms of toxicity, and impacts at environmentally relevant concentrations. Therefore, the continuous and increasing production, use, and release of CAF into the environment could result in potential sublethal and long-term ecological risks for aquatic biota. Thus, further studies are crucial to identify the potential repercussions of CAF exposure in different aquatic ecosystems, with varying levels of contamination and environmental relevance. More multifaceted analyses involving various aquatic organisms, occupying key positions in the food web, are essential to generate ecological, physiological, toxicological, evolutionary, epigenetic, and genetic data for a better understanding of the effects of CAF on aquatic ecosystems. The results of this study also raise awareness in the scientific community and society in general about the environmental contamination issues and the sustainability and safety of ecosystems associated with the CAF levels detected in surface waters.

## Data Availability

No datasets were generated or analysed during the current study.
